# Tuning the surface functionality of polyethylene glycol-modified graphene oxide/chitosan composite for efficient removal of dye

**DOI:** 10.1038/s41598-023-40701-9

**Published:** 2023-08-18

**Authors:** Md. Nahid Pervez, Md Anwar Jahid, Mst. Monira Rahman Mishu, Md Eman Talukder, Antonio Buonerba, Tao Jiang, Yanna Liang, Shuai Tang, Yaping Zhao, Guilherme L. Dotto, Yingjie Cai, Vincenzo Naddeo

**Affiliations:** 1 Hubei Provincial Engineering Laboratory for Clean Production and High Value Utilization of Bio-Based Textile Materials, Wuhan Textile University, Wuhan, 430200 China; 2 Sanitary Environmental Engineering Division (SEED), Department of Civil Engineering, University of Salerno, via Giovanni Paolo II 132, 84084 Fisciano (SA), Italy; 3 Department of Chemistry and Biology “Adolfo Zambelli”, University of Salerno, via Giovanni Paolo II, 84084 Fisciano, Italy; 4 Department of Environmental and Sustainable Engineering, University at Albany, State University of New York, Albany, NY 12222 USA; 5 Shanghai Engineering Research Center of Biotransformation of Organic Solid Waste, School of Ecological and Environmental Sciences, East China Normal University, and Institute of Eco-Chongming, Shanghai, 200241 China; 6 Research Group on Adsorptive and Catalytic Process Engineering (ENGEPAC), Federal University of Santa Maria, Av. Roraima, 1000-7, Santa Maria, RS 97105-900 Brazil

**Keywords:** Pollution remediation, Materials science

## Abstract

There has been a lot of attention on water pollution by dyes in recent years because of their serious toxicological implications on human health and the environment. Therefore, the current study presented a novel polyethylene glycol-functionalized graphene oxide/chitosan composite (PEG-GO/CS) to remove dyes from aqueous solutions. Several characterization techniques, such as SEM, TEM, FTIR, TGA/DTG, XRD, and XPS, were employed to correlate the structure–property relationship between the adsorption performance and PEG-GO/CS composites. Taguchi’s (L_25_) approach was used to optimize the batch adsorption process variables [pH, contact time, adsorbent dose, and initial concentration of methyl orange (MO)] for maximal adsorption capacity. pH = 2, contact time = 90 min, adsorbent dose = 10 mg/10 mL, and MO initial concentration = 200 mg/L were found to be optimal. The material has a maximum adsorption capacity of 271 mg/g for MO at room temperature. With the greatest R^2^ = 0.8930 values, the Langmuir isotherm model was shown to be the most appropriate. Compared to the pseudo-first-order model (R^2^ = 0.9685), the pseudo-second-order model (R^2^ = 0.9707) better fits the kinetic data. Electrostatic interactions were the dominant mechanism underlying MO sorption onto the PEG/GO-CS composite. The as-synthesized composite was reusable for up to three adsorption cycles. Thus, the PEG/GO-CS composite fabricated through a simple procedure may remove MO and other similar organic dyes in real contaminated water.

## Introduction

Recent years have seen a rapid growth of urban industrialization and modernization, leading to the generation of a large amount of industrial wastewater effluent^1–3^. Unfortunately, directly discharging these effluents into the environment is not sustainable, in which natural water bodies are polluted with organic and inorganic pollutants. Among them, organic dyes are considered one of the most toxic because of their devastating effects on the environment and the human body when it is inhaled or ingested^4–6^. As a result, developing efficient, economical, and environmentally friendly dyes removal technology has become a collaborative objective during the past few decades.

Several methods, such as membrane filtration, coagulation/flocculation, adsorption, Fenton oxidation, and so on, have been used in dye removal from wastewater. As a result of its low price, high efficiency, and widespread use, adsorption has become the dominant technological approach. It excels at removing various pollutants from wastewater discharged by industries^7,8^. Till now, there has been widespread interest in graphene oxide (GO), a typical carbonaceous substance frequently generated by the chemical oxidation of graphite. With its vast surface area and abundance of oxygen-containing functional groups, GO has been shown to be an effective adsorbent for organic dyes^9^. Because of its distinctive architecture and electrical characteristics, GO can engage in powerful interactions with dyes and organic molecules in the form of non-covalent bonds. These interactions may occur through hydrogen bonding, atomic stacking electrostatic, and van der Waals forces^10^. However, some drawbacks of GO that limit its feasible applications include tiny sizes, dispersing in water, and creating a robust colloidal solution, making it difficult to separate and recycle^11^. Besides, in view of the strong anionic charge of GO, there is much lower adsorption anionic than cationic pollutants. Blending in cationic-charged natural polymers would be a sustainable choice with a theoretically improved adsorption capacity to overcome this.

Chitosan (CS) is a natural cationic polysaccharide from chitin's deacetylation. Because it contains amine and hydroxyl groups, which can function as chelating and reaction sites, it can physically and chemically entrap various metal ions^12,13^. In recent years, chitosan has piqued the attention of many scientists owing to its remarkable features. Especially structural reactivity, biodegradability, and biocompatibility make it an excellent choice for a variety of environmental remediation applications^14,15^. Previous studies documented that adding CS to GO improved the overall GO/CS composite-specific surface sites and dispersion properties by forming a crosslinking reaction, thus promoting their adsorption performance towards dye molecules. For example, Travlou et al.^16^ prepared a GO/CS composite-based adsorbent, and the adsorption behaviour of Reactive Black 5 dye was found to be 277 mg/g. A recent study by Tran et al.^17^ reported that GO crosslinked CS composites could be an efficient adsorbent for methylene blue (MB) (maximum adsorption capacity was 259.5 mg/g). Nevertheless, these composites often exhibited low specific adsorption capacity toward target molecules and required long contact time to reach equilibrium owing to the unavoidable competition for the accessible adsorption sites from other organic and metallic contaminants^18^. Accordingly, several studies reported that the maximum adsorption capacity of dye molecules by the GO/CS composite was lower than the modified GO/CS composite. For example, β-cyclodextrin/chitosan functionalized graphene oxide hydrogel had a sorption capacity of 1134 mg/g for MB)^19^, and EDTA-chitosan functionalized magnetic graphene oxide adsorbed Rhodamine B at 1085.3 mg/g^20^. Thus, additional GO/CS composite adjustments are needed to enhance its adsorption capacity for dye molecules.

In water treatment, polyethylene glycol (PEG) is considered a useful additive by virtue of its thermosensitivity, mechanical and chemical stability, water solubility, and non-toxicity. Adding PEG to composites may greatly enhance their biocompatibility. PEG has been employed frequently in the process of surface-modifying composites, which are applied to treat wastewater. For example, the PEG-loaded alginate-Zr^4+^ network was synthesized by Luo et al.^21^, in which they achieved a better phosphorus adsorption trend due to a microporous structure. The presence of PEG may have tuned the physicochemical properties, which boosted the exposure of active adsorption sites and increased phosphate dispersion into the beads' interiors. Similarly, Mandal et al.^22^ developed PEG-modified Layered Double Hydroxides-based adsorbents and found that the adsorption capacity of Orange II was increased by ~ 30% compared to the pristine Layered Double Hydroxide. PEG modification has been proven as an effective approach to modulating the adsorption efficiency. As a result, it is clear that adding the PEG modifier to the GO/CS surface is a potential strategy to improve GO/CS adsorptive performance; having different functional groups might play an important role in interacting with dye molecules^23^. Moreover, the application of PEG-functionalized CS/GO composite has not yet been reported for environmental remediation, which is of core interest in the present study.

In general, the adsorption processes are impacted by multiple factors, such as pollutants concentration, time, dose, and pH of the solution; therefore, a systematic approach to the design, execution, and assessment of the process is required in order to accomplish the maximal removal of the pollutant. Traditional optimization techniques that adjust only one parameter while keeping all other parameters the same are often thought to be time- and labor-intensive. In contrast, the design of experiments (DOE), sometimes referred to as a systematic approach, is a method that seeks to establish a connection between factors affecting a process and its outcome. Full factorial design and Taguchi experimental design are two general divisions of the DOE technique. The complete factorial design evaluates and analyzes every conceivable combination of parameter values. In comparison, in Taguchi's experimental design research, only selected levels are evaluated. The Taguchi approach is durable as it employs an orthogonal array (OA) design. The OA can quantitatively determine the appropriate parameters and levels, and it is used to cut down on the number of trials, the length of tests, the expense, and the quantity of labor-intensive work needed.

The present work investigates the adsorption performance of methyl orange using PEG-functionalized GO/CS composites via the batch process, which is the first time reported in the literature. Subsequently, the physicochemical properties were systematically investigated using various tools, such as SEM, TEM, FTIR, XRD, TGA, and XPS. After that, MO adsorption studies were carried out in a batch mode, and the effects of operational parameters, such as pH, time, dosage, and dye concentration, were optimized through the Taguchi design. Besides, the effect of inorganic anions and reusability tests were examined under the optimized condition. Finally, a tentative adsorption mechanism was described to gain better insights into adsorption.

## Experimental

### Materials and reagents

Commercial-grade chitosan powder was obtained from Shandong Chitin Powder Factory (Shandong, China). It has a viscosity-average molecular weight of 600,000 g/mol and a deacetylation degree of 95%. The graphene oxide (GO) powder form was bought from Jiangsu XFNANO Materials Tech Co., Ltd (China). Polyethylene glycol (PEG, molecular weight of 2000 g/mol) was purchased from Alfa Aesar (USA). Methyl orange (MO, molecular weight of 327.33 g/mol) was obtained from CARLO ERBA Reagents s.r.l. Milan, Italy. The remaining reagents were of analytical quality and did not need further purification before use.

### Preparation of adsorbents

**Figure 1:**
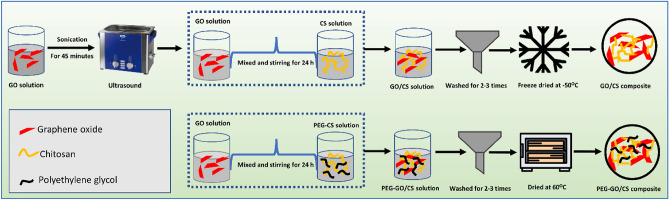
The preparation steps of graphene oxide (GO)/chitosan (CS) and polyethylene glycol functionalized (PEG-GO/CS) composite.

The GO/CS composites were prepared as follows: a uniform suspension of GO solution was obtained by dissolving 0.2 g of GO into 50 mL of ultrapure water and then subjecting the mixture to 45 min of treatment in an ultrasonic bath. After that, 19.8 g CS was dispersed in 1980 mL of acetic acid and added to the GO solution, followed by stirring for 24 h^24^. Then, the final composite of GO/CS (1 wt%) was achieved by freeze-drying at − 50 °C for 48 h. In the case of 1.5 wt% GO/CS/PEG composite, a known amount of GO (0.3 g) and 19.4 g CS in 1920 mL acetic acid (contained 0.3 g PEG) were mixed and stirred for 24 h. After that, the GO/CS/PEG mixture was brought to room temperature and sonicated for 5 min to remove any bubbles that had formed and 2–3 times washed using distilled water. Finally, solid samples were made by drying the liquid samples for 24 h at 60 °C. Similarly, 2 wt% GO/CS/PEG composite was prepared by mixing a known amount of GO (0.4 g) and 19.2 g chitosan in 1900 mL acetic acid containing 0.4 g PEG. The preparation steps are shown in Fig. 1.

### Characterization

#### Scanning electron microscope

A field-emission scanning electron microscope (Philips SEM 515, Germany) was utilized in order to investigate the surface morphologies of as-prepared samples. The samples were covered with gold before the measurements, and then they were subjected to an accelerated voltage of 10 kV for observation.

#### Transmission electron microscopy

Transmission electron microscopy (TEM, JEM-2100, Japan) with an acceleration voltage of 80–200 kV was used to ascertain the structure and orientation of the synthesized composites. The samples were assessed at a magnification of 50–1,500,000, a spot resolution of 0.23 nm, and a lattice resolution of 0.14 nm.

#### X-ray photoelectron spectroscopy

Using an X-ray photoelectron spectrometer, the surface chemical compositions of the samples were examined (ThermoFisher ESCALAB 250 XI). AlKα, with a pass energy of 20 eV, served as the X-ray source (h = 1360 eV). The XPS chamber was under 300 W of power, and the pressure varied from 107 to 109 Pa.

#### FTIR analysis

A Bruker Optik EQUINOX 55 spectrophotometer (Ettlingen, Germany) was used to analyze the Fourier Transformation Infrared (FTIR) spectra of samples over the wavenumber range of 4000–400 cm^−1^. After cutting and mixing the samples with the potassium bromide, the samples were ground into a pellet, which was then kept at room temperature (23 °C).

#### XRD analysis

Powder XRD patterns of samples were analyzed using a Rigaku Ultima III X-ray diffractometer (Texas, USA). Using CuKα radiation with a wavelength of λ = 1.54056 as the source, the crystallization behavior configuration was scanned from a phase angle of 2θ = 5°–60° with a step size of 0.02°.

#### Thermogravimetric analysis

A thermogravimetric analyzer (TGA/DSC1, Mettler-Toledo Instruments Co., Ltd. Shanghai, China) was used to investigate the thermal behaviour of samples. The curves were measured from 30 to 800 °C at a constant heating rate of 10 °C/min in a nitrogen environment (flow rate of 50 mL/min).

#### Zeta potential

The adsorbent's surface charge through zeta potential was measured employing a Nano series ZETA SIZER (PHENOM-WORLD, Netherlands). Using a homogenizer, the samples were added to the deionized water to keep the concentration at 10 g/L and maintained pressure around 20,000 Pa under room temperature.

### Batch adsorption

Using VWR centrifuge polypropylene tubes, 10 mL of a 200 mg/L MO solution was added to a total of 10 mg of each adsorbent (GO/CS, 1.5% PEG-GO/CS, and 2% PEG-GO/CS) and magnetically agitated at 200 rpm for 2 h at neutral pH and room temperature to determine the most effective adsorbent. During each experiment, a certain volume of the solution was withdrawn at regular intervals and filtered using a 0.45 m membrane filter. The amount of MO was evaluated at a wavelength of 467 nm using a Perkin-Elmer Lambda 25 UV–Vis spectrophotometer (Waltham, MA, USA). To determine the percentage of removal, the following Eq. (1) was used:

1 $$ \% \; Removal = \frac{{C_{0} - C_{e} }}{{C_{0} }} \times 100 $$

where C_0_ (mg/L) represents the initial MO concentration, C_e_ (mg/L) denotes the concentration at equilibrium.

Based on the removal %, the 1.5% PEG-GO/CS adsorbent was selected for future investigation. It has been observed that the impact of controllable parameters on adsorption capacity was significant, and because of this, it is worthwhile to analyze it in further detail using the Taguchi analysis optimization approach. This investigation examined four factors, including contact duration, pH, adsorbent dose, and starting concentration of MO, and five different levels of each factor were tested (Table 1). In each trial, 10 mL of dye solution was stirred at 200 rpm. By periodically adding either 0.1 mol HNO_3_ or 0.1 mol NaOH solutions, the pH of the solution was maintained in the desired 2–9 range. According to Table 1, adsorption studies in the batch mode were carried out at room temperature. The adsorption capacity was determined using the below Eq. (2):

2 $$ Q_{e} = \frac{{V \times (C_{0} - C_{e} )}}{m} $$

where Q_e_ (mg/g) is the adsorption amounts at equilibrium, C_e_ (mg/L) denotes the concentration at equilibrium, C_0_ (mg/L) denotes the initial MO concentration, m (g) is the dosage of the adsorbent, and V (L) is the adsorbed solution volume.

**Table 1:** Selection of factors and levels in the Taguchi design.

Symbol	Factors	units	Level 1	Level 2	Level 3	Level 4	Level 5
A	pH	–	2	4	7	8	10
B	Contact time	min	5	20	35	60	90
C	Adsorbent dose (/10 mL)	mg	2	4	6	8	10
D	Initial dye concentration	mg/L	50	100	150	200	250

Adsorption isotherms were examined by adding a known quantity of 1.5% PEG-GO/CS (10 mg) to 10 mL solutions of 50–250 mg/L MO in a series of conical flasks. The resultant mixture was shaken for an hour in a water bath maintained at room temperature. The solution was filtered at the end, and the resulting filtrate's MO content was measured spectrophotometrically at 467 nm.

The batch adsorption studies were performed in a series of conical flasks, allowing for the measurement of the adsorption process' kinetic characteristics. At a pH of 2, each flask has 10 mL of MO solution (200 mg/L) and 10 mg of adsorbent in it. Each flask's contents were agitated in a shaker bath at room temperature for a fixed amount of time. After that, the shaker was turned off, and the flasks were filtered. Absorbance measurements at 467 nm were used to determine the remaining MO concentration in the filtrate after the process. Adsorption capabilities were determined at various times and then fit into kinetic models.

An aqueous solution of 200 mg/L MO dye and varying concentrations of NaCl (0.1–0.5 M) was used to study the impact of ionic strength on the adsorption capacity of MO. To carry out the regeneration test, 10 mg of adsorbent was added to 10 mL MO solutions (200 mg/L) in a conical tube. The adsorbent was separated, rinsed with distilled water, and placed in a 50 mL conical tube after 2 h of agitation at 200 rpm. After the addition of 10 mL of the 0.05 M NaOH, the mixture was agitated for 2 h. The adsorbent was removed, washed, neutralized, and reintroduced to the fresh MO solution before being removed, washed, and neutralized. In order to test the produced adsorbent's reusability, three cycles of regeneration and reuse were performed.

## Results and discussion

### Characterization

**Figure 2:**
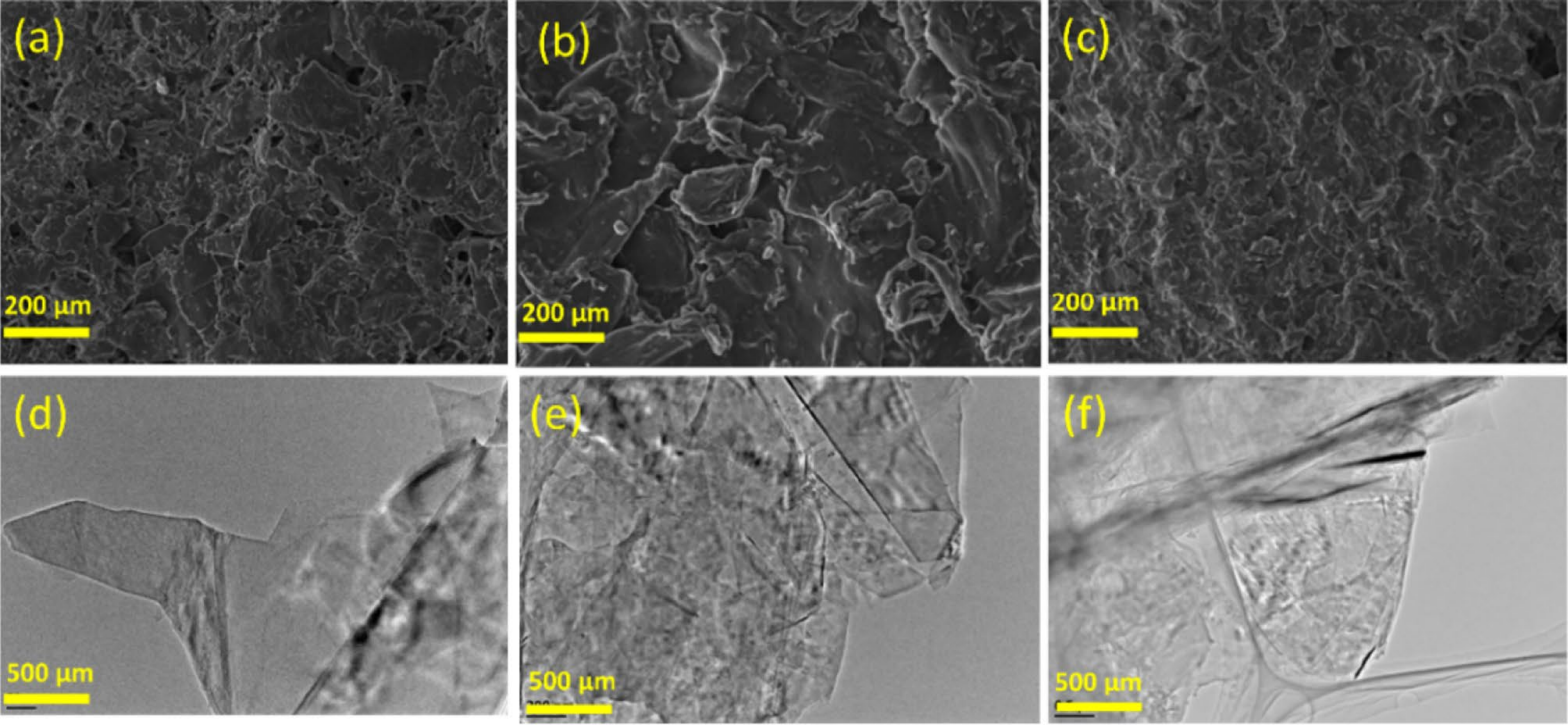
SEM and TEM images of 1% GO/CS (**a**,**d**), 1.5% PEG-GO/CS (**b**,**e**), and 2% PEG-GO/CS (**c**,**f**), respectively.

The application of functionalization would profoundly change the substrate material's form and the fundamental characteristics of its surfaces. We examined the surface characteristics and interfacial interactions of GO/CS and the GO/CS/PEG composites using SEM and TEM images. As seen in Fig. 2a,d, the surface of the GO becomes rougher after being grafted with CS, indicating the GO's crosslinking or tangling with CS. The GO sheets are disseminated in the chitosan matrix in a unidirectional manner parallel to the composite (Shadow of the thin film). Establishing amide connections between GO and chitosan can make it easier to disperse GO throughout the chitosan matrix^25^. The subsequent PEG-modified GO/CS composite surface results in an even greater surface roughness increase (Fig. 2b,e). A 3D network structure was formed when the molecular chain of PEG was incorporated, which might further decrease the van der Waals force between molecules and enhance the dispersion of GO^26^. Additionally, as can be observed in Fig. 2c,f, the PEG-GO/CS composite structure is more fracture and irregular due to the higher concentration. Overall, according to SEM and TEM analysis, 1.5% PEG-GO/CS sample is accompanied by distinctive morphology with a more stable surface, which is advantageous for applications involving the trapping of dye substances.

**Figure 3:**
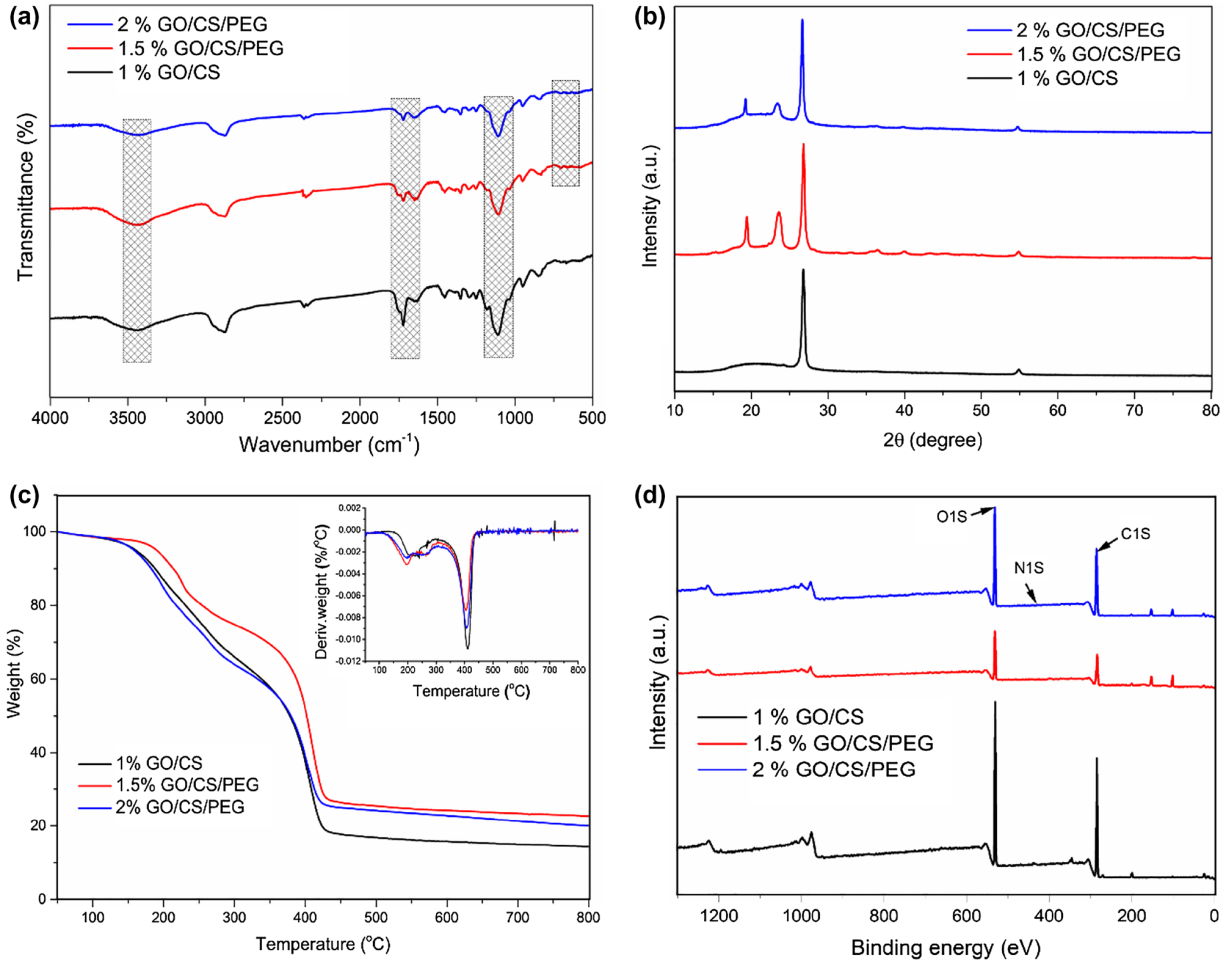
FTIR (**a**), XRD (**b**), TGA and DTG (**c**) and XPS (**d**) of 1% GO/CS, 1.5% PEG-GO/CS, and 2% PEG-GO/CS, respectively.

The interaction of functional groups between GO/CS and GO/CS/PEG composites was investigated using a Fourier transform infrared spectroscopy (FTIR), and the spectra are presented in Fig. 3a. The FTIR spectra of GO/CS reveal the combination characteristics of pure graphene oxide and chitosan, which includes the broad peak at 3424 cm^−1^ corresponding to the mixture of amine stretch (CS) and OH groups (GO). Next, the peak at 1688 cm^−1^ demonstrates the existence of COOH groups derived from graphene oxide, which is moved lower due to hydrogen bonding between GO and the hexatomic ring of CS^27^. The peak at 1528 cm^−1^ certifies C=C groups in GO, indicating the existence of N–H bonding due to the presence of CS. The peak around 1098 cm^−1^ due to C–O–C stretching can be associated with GO layers. Most importantly, the amide interaction between the carboxylic in GO and NH_2_ groups in CS is shown by the disappearance of the carboxylic acid peak^28^ at 1731 cm^−1^. The data suggested that GO and CS interacted with one another. The FTIR spectra of PEG-GO/CS displayed considerable similarities to those of GO/CS and PEG, and To that end, the PEG modifier is characterized by a set of spectral features, including stretching vibrations (C–H) at 2960–2880 cm^−1^ and stretching (C–O–C) found between 1250–1030 cm^−1^. Moreover, the OH groups stretching of the PEG causes a strong and sharp peak at a higher wavenumber^29^ of 3435 cm^−1^. Besides, the peak at 1821 cm^−1^ generalized the ester bonding confirmation between PEG and GO/CS sheets. Finally, the 832 and 952 cm^−1^ peaks represent PEG's crystalline characteristics, which can demonstrate the successful composite formation of PEG-GO/CS.

Figure 3b displays the XRD patterns for GO/CS, 1.5% PEG-GO/CS, and 2% PEG-GO/CS from 10° to 80°. An amorphous structure may be inferred from the XRD pattern of GO/CS, which displays a strong peak at 2θ = 25.8°. The disappearance of the typical CS diffraction peaks and the GO diffraction peak is intriguing since it shows that the fully exfoliated arrangement of GO sheets has been established in the chitosan structure^24^. On the other hand, two peaks at 2θ = 19.48° and 2θ = 23.43° characteristic of PEG^30^ can be seen as a result of PEG's integration into the GO/CS composite. When comparing the 2% PEG-GO/CS to the 1.5% PEG-GO/CS sample, it is clear that the latter has more crystallinity tuning flexibility. Overall, the crystallinity of the composite improves once PEG is added due to the high crystalline structure of PEG molecules themselves^31^.

As seen in Fig. 3c, the thermogravimetric analysis (TGA) was used to explain the thermal stability of GO/CS, 1.5% PEG-GO/CS, and 2% PEG-GO/CS. The TGA is useful for analyzing a sample's weight loss due to heating it in a certain environment. Differential thermogravimetric analysis, often known as DTG (inset of Fig. 3c), was used to identify the temperature at which the highest rate of weight loss would occur, and this value was found to be precisely the same as the breakdown temperature. Figure 3c shows that the GO/CS sample had three distinct loss phases between 50–100 °C, 150–330 °C, and 350–430 °C. This is in line with the loss of water, the breakdown of oxygenated functional groups, and the complete pyrolysis of the carbon skeleton^32^. Figure 3c shows a thermogram developed with 2% PEG-GO/CS, which is identical to GO/CS except that the beginning decomposition temperature of the composite has moved to about 180 °C and the weight loss occurring during 350–430 °C has risen from 19% (GO/CS) to 22.0% (1.5% PEG-GO/CS). Figure 3c shows that 1.5% PEG-GO/CS degradation begins at 198 °C, with intermediate temperatures of 150–330 °C and 350–430 °C, much as GO/CS and 2% PEG-GO/CS. Weight loss is minimal compared to GO/CS and just 2% in PEG-GO/CS degradation steps. One possible explanation is that PEG bonds with the GO/CS combination^33^. Because of the strengthened connections between the PEG and the GO/CS, the composite can withstand temperatures up to 800 °C without losing more than 30% of its initial weight. Given this increased stability, the PEG-loaded GO/CS composite seems to be a good fit for high-temperature-aided water treatment.

XPS spectra verified the surface composition of 1% GO/CS, 1.5% PEG-GO/CS, and 2% PEG-GO/CS (Fig. 3d). The XPS survey spectra indicate three peaks at around 285.2 eV, 400.5 eV, and 532.7 eV, assigned to the C1s, N1 s, and O1s core levels, respectively; their narrow scan spectra are shown in Fig. S1. Table S1 provides an overview of binding energy and atomic surface concentration. According to the relative elemental analysis, the PEG-GO/CS composite had higher oxygen and nitrogen levels than GO/CS but lower carbon content^34^. Consistent with the FTIR findings, PEG-GO/CS was highly soluble in an aqueous solution due to several hydrophilic functional groups on its surface. In producing GO/CS-PEG composites, the hydrophilic ligand, such as PEG, may play an important role.

### Selection of adsorbent

**Figure 4:**
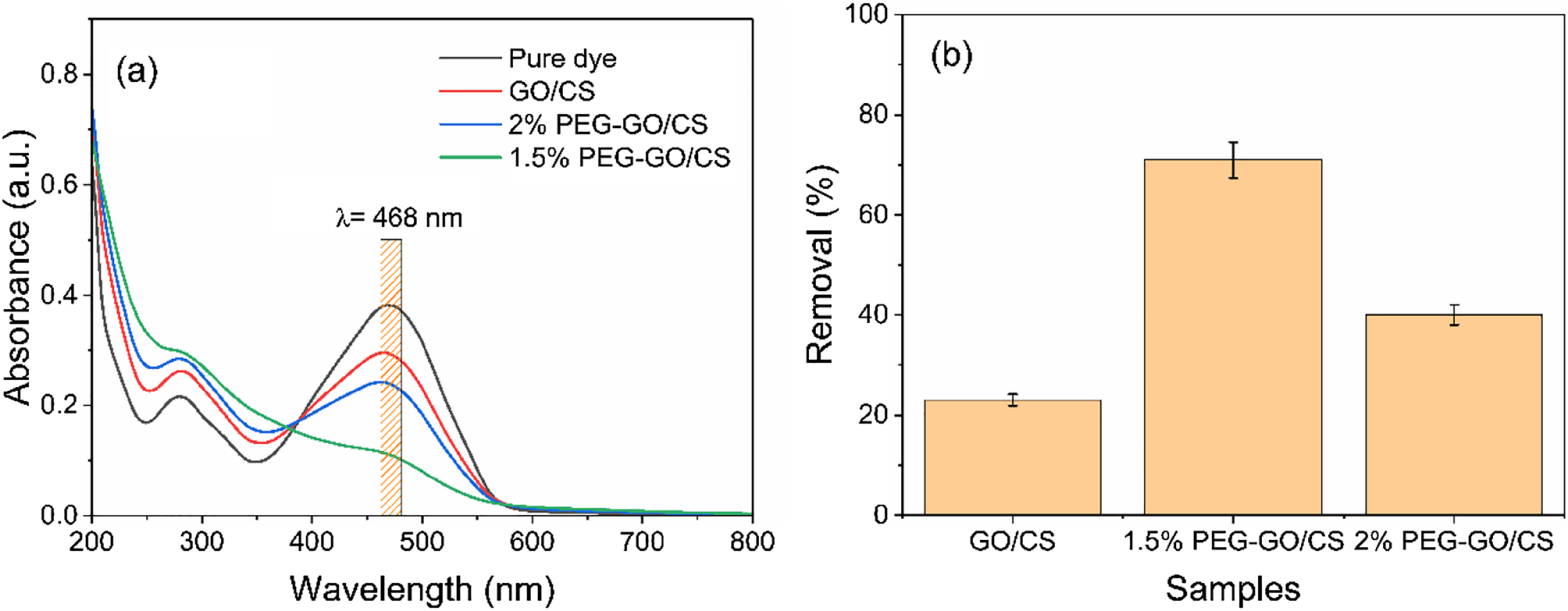
UV–Vis spectra of dye removal (**a**) and removal % (**b**) by 1% GO/CS, 1.5% PEG-GO/CS, and 2% PEG-GO/CS, respectively.

The MO was used as a model synthetic dye. The removal efficiency of several adsorbents synthesized under different circumstances was studied to determine which adsorbent was most suited for the batch tests. The relative effectiveness of several adsorbents in removing MO is shown in Fig. 4a,b. The removal efficiency of MO varies greatly amongst adsorbents. In the case of GO/CS, its MO-removal capacity is just 23%. When PEG was added to the GO/CS composite, a much higher removal of MO was found.

Regarding the 2% PEG-GO/CS ratio, the MO removal percentage was only 40%. On the other hand, 1.5% PEG-GO/CS enhanced the removal of the MO to 71%. This may be ascribed to the fact that introducing PEG at this dose offers additional adsorption sites and a higher positive surface charge (Fig. S2), leading to increased MO sorption. Therefore, we choose the 1.5% PEG-GO/CS composite for further adsorption studies.

### Taguchi analysis of Qe of 1.5% PEG-GO/CS

Adsorption of MO was carried out using the L25 orthogonal experimental technique, followed by the determination of optimal sorption condition using the 1.5% PEG-GO/CS. Table 2 displays the Qe values concerning their S/N values for the adsorbed samples. In general, the more dye adsorbed as an adsorbate, the greater the Qe of the sample.

**Table 2:** Results of MO adsorption based on L_25_ Taguchi design.

Experiments no	Parameters				Qe (mg/g)	S/N
	A	B	C	D		
1	2	5	2	50	39.06	31.83
2	2	20	4	100	62.39	35.90
3	2	35	6	150	90.72	39.15
4	2	60	8	200	141.20	42.99
5	2	90	10	250	135.48	42.63
6	4	5	4	150	80.34	38.09
7	4	20	6	200	130.12	42.28
8	4	35	8	250	128.21	42.15
9	4	60	10	50	40.12	32.06
10	4	90	2	100	72.12	37.16
11	7	5	6	250	128.43	42.17
12	7	20	8	50	30.09	29.56
13	7	35	10	100	72.21	37.17
14	7	60	2	150	81.78	38.25
15	7	90	4	200	128.32	42.16
16	8	5	8	100	52.12	34.34
17	8	20	10	150	87.23	38.81
18	8	35	2	200	118.32	41.46
19	8	60	4	250	114.21	41.15
20	8	90	6	50	25.21	28.03
21	10	5	10	200	108.32	40.69
22	10	20	2	250	101.78	40.15
23	10	35	4	50	22.12	26.89
24	10	60	6	100	42.17	32.50
25	10	90	8	150	88.23	38.91

#### Signal-to-noise (S/N) ratio assessment

The fundamental goal of Taguchi's study, which often involves the S/N ratio, was to determine the optimal environment for acquiring and analyzing the different processing parameters. The S/N ratio is used to evaluate the degree to which observed data deviates from the predicted one, where signal stands for desired values and noise for unexpected ones. There are three different S/N ratios, and for the sake of this investigation, the one with the largest sample size was chosen (Eq. 3)^35^ (higher adsorption capacity).

3 $$ {\text{S/N}} = - 10 \log \left( {\frac{1}{{\text{n}}}\mathop \sum \limits_{{{\text{i}} = 1}}^{{\text{n}}} \frac{1}{{{\text{y}}_{{\text{i}}}^{2} }}} \right) $$

where y_i_ is the result of the ith experiment in the orthogonal array design, and n is the total number of times the experiment was performed. Table 3 displays the response average S/N values derived at the 5 levels for the 4 factors. We also calculated delta characteristics to identify the most critical parameters by subtracting the highest and lowest average S/N ratio values from each set^36^. Then, they're assigned using a rating system where 1 represents the highest value, 2 is the next highest, 3 is the next highest, and so on. The highest delta value (12.24) was found for the initial dye concentration, making it clear that this was the most essential element. The next three most important factors were solution pH (2.67), adsorbent dose, and time, with a value of 1.45 and 0.44, respectively.

**Table 3:** Response table for S/N ratios in Qe of MO adsorption.

Level	A	B	C	D
1	38.51	37.43	37.77	29.68
2	38.35	37.34	36.84	35.42
3	37.87	37.37	36.83	38.65
4	36.76	37.39	37.60	41.92
5	35.83	37.78	38.28	41.66
Delta	2.67	0.44	1.45	12.24
Rank	2	4	3	1

**Figure 5:**
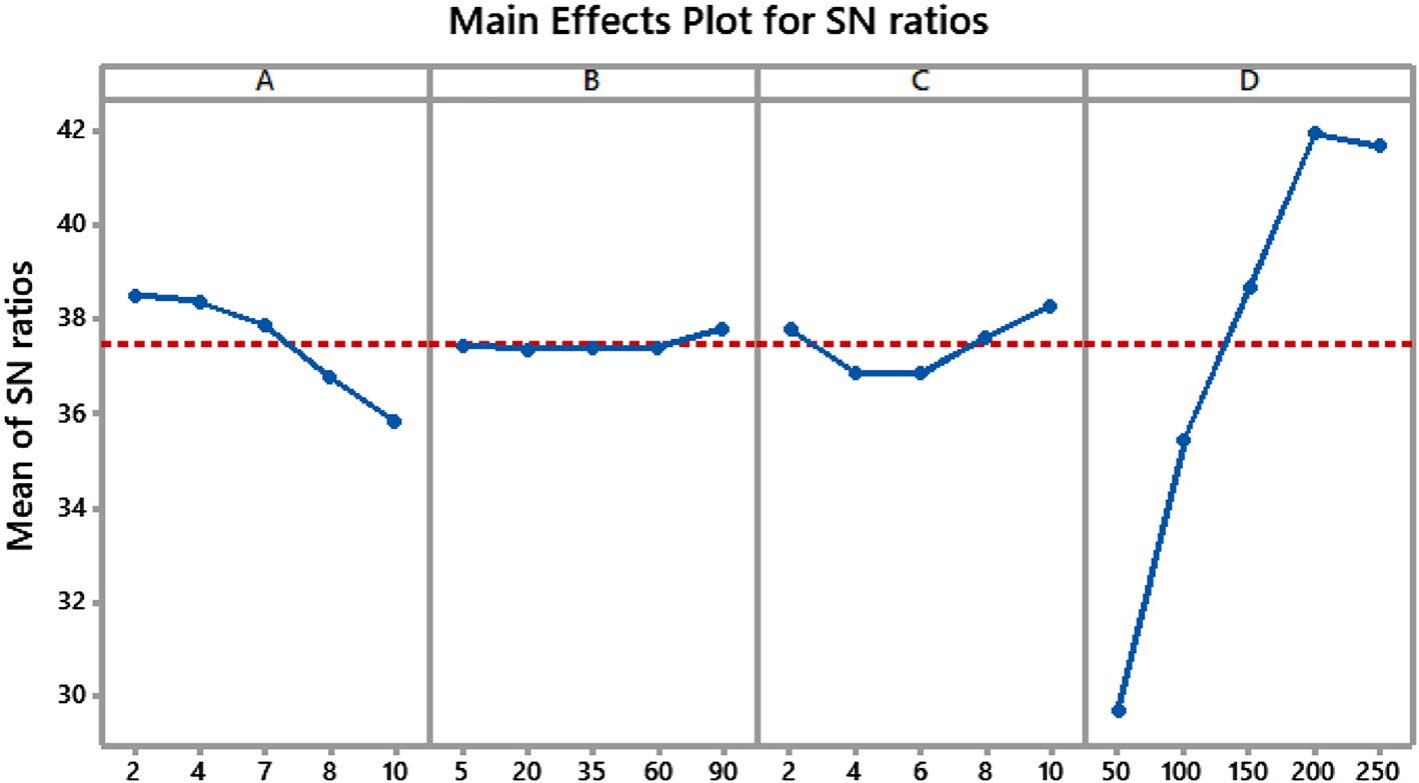
Main effects plot for S/N ratios in Qe of MO adsorption.

In addition, the major impact plot of the process parameters for the S/N ratios (data means) in Qe of MO adsorption is shown in Fig. 5. The effect of the different process parameters may be shown by comparing the values of the separate process parameters to a solid line. If a certain process parameter is located close to the dashed line, this suggests that the process only has little impact on adsorption behavior. On the other hand, the adsorption process is predominantly influenced by a parameter with a steeper slope. Thus, among the factors that were investigated, it was found that initial dye concentration (D) and solution pH (A) had a statistically significant effect on adsorption capacity. On the other hand, adsorbent dose (C) and time (B) had a very moderate impact. Considering this, the best circumstances have been classified as A1B5C5D4, which resulted in the greatest possible Qe when the Taguchi approach was used. This is significant since the highest possible Qe signifies the most effective adsorption performance.

#### Interaction plots

**Figure 6:**
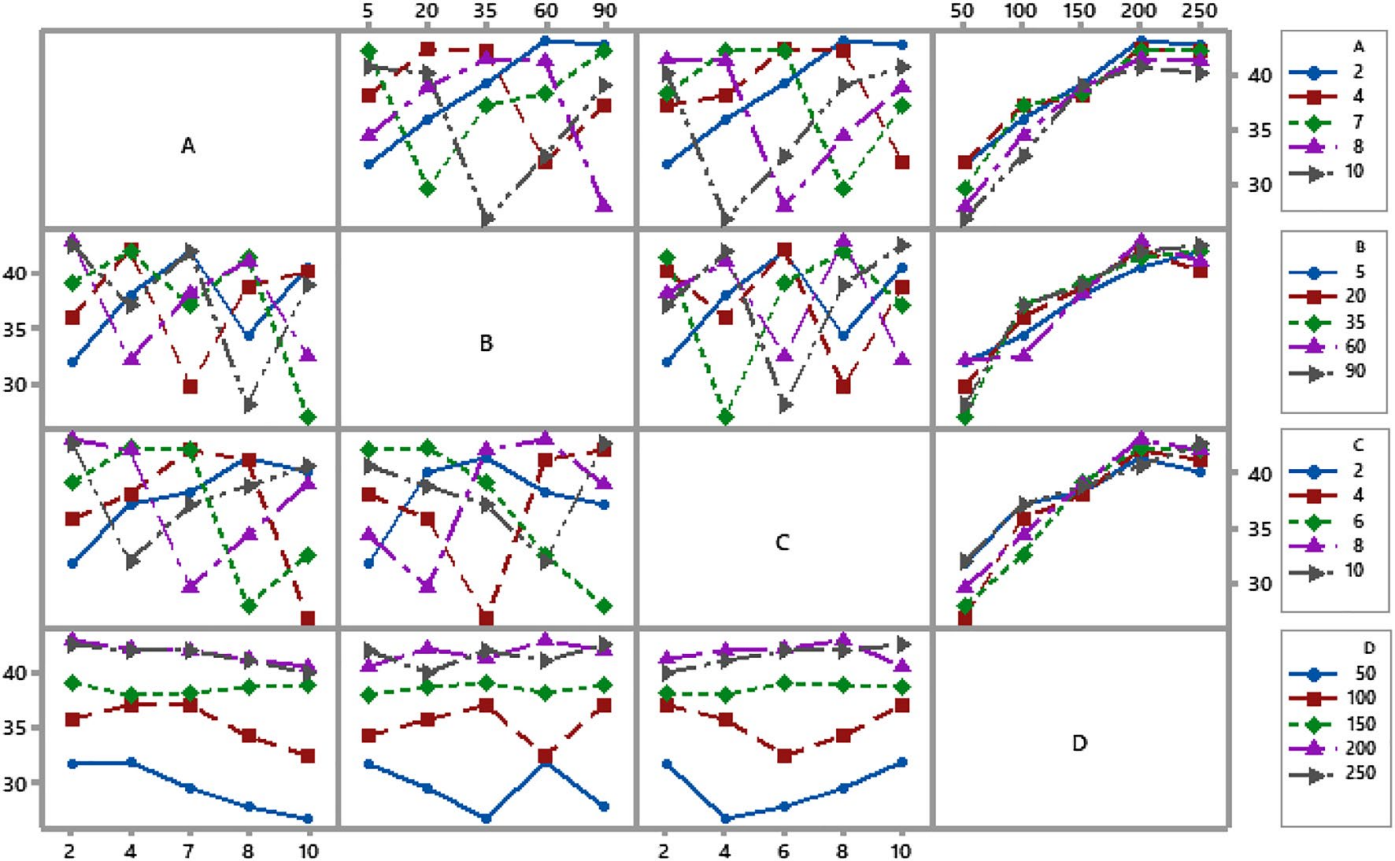
Full interaction plot for S/N ratios (Qe).

The behavior of the levels of process parameters depends on their interaction, which can only be determined by analyzing the interaction graph. Two distinct patterns of behavior resulting from interactions are possible: parallelism and non-parallelism. The interaction effects of the input parameters may be seen by examining the plot's non-parallel and parallel lines. Parameters are highly dependent on one another for non-parallel lines but only somewhat dependent in the case of parallel lines^37,38^. Figure 6 shows the interaction plot, which shows that the three independent variables (adsorbent dose, contact time, and solution pH) have significant interactions, as shown by the non-parallel lines (three lines intersect with each other). Besides, it was observed that the initial dye concentration (D) had non-parallel solid lines, indicating that its values were not as reliant on each other as was initially assumed, leading to the most influential parameter. Overall, analysis of interaction plots, which are very useful for analyzing process aspects, reveals that the selected parameters had a major effect on the methyl orange adsorption efficiency.

#### Analysis of variance (ANOVA)

**Figure 7:**
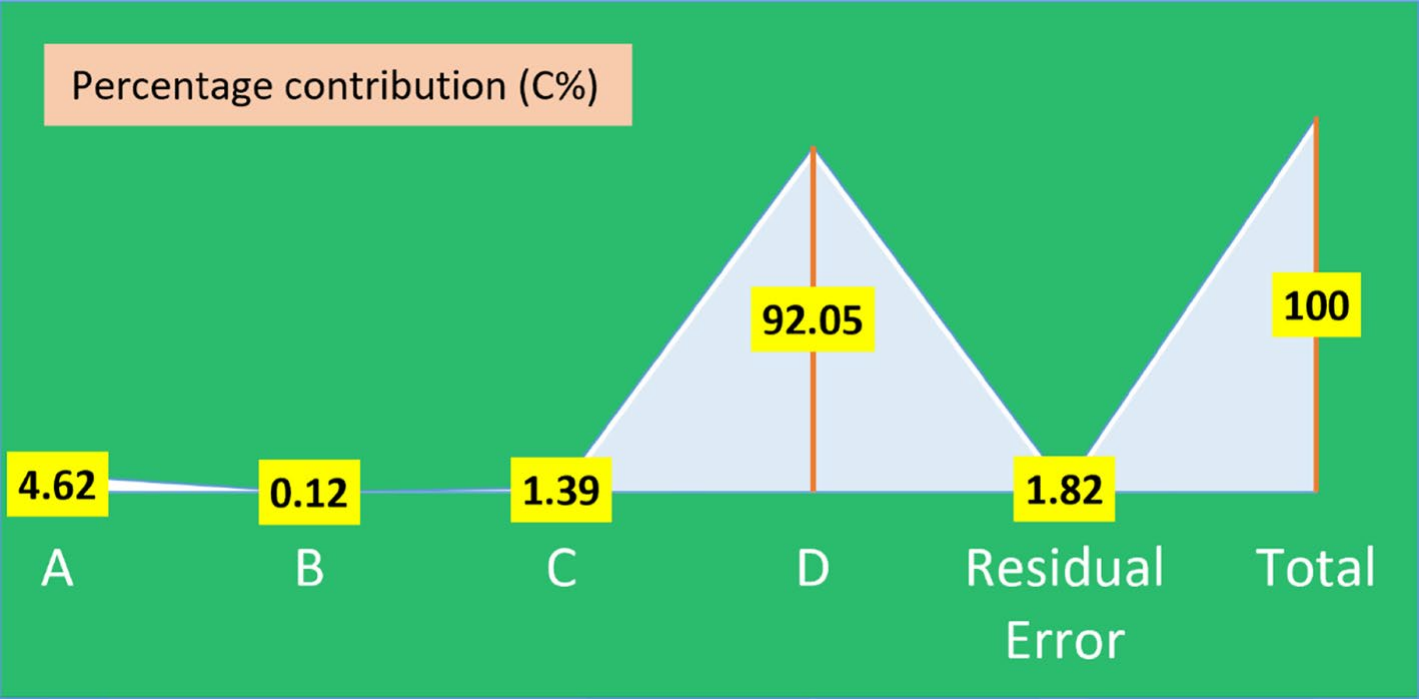
Percentage contribution (%) of each parameter.

After applying the Taguchi technique to the findings gained from experimental testing and the calculated S/N ratio, an ANOVA is used to determine the relative relevance of different components. The S/N ratio may be used to determine the optimum conditions in which the maximum response value is obvious. However, it cannot provide data on the central variable in the experiment, whereas ANOVA is able to exhibit the required information. In this case, the SS factors produced from trial results were initially supposed to be used to split the distribution of measurements. The proportion of MS values was utilized for F-value since it represents the most critical factor in the model. If the p-value of a factor is less than 0.05, it is deemed significant^39,40^. ANOVA results for Qe of MO are shown in Table 4. The greatest F-value found for this relationship was 152.17, with a corresponding p-value of 0.00 (significant) and an overall contribution percentage as high as 92.05% (Fig. 7), entirely attributable to the initial dye concentration. Also, the solution pH showed statistical significance (0.02) with a percentage contribution (C%) of 4.62 (Fig. 7). Moreover, the percentage contributions from adsorbent dose and contact time, respectively, were 1.39% and 0.12%, with the latter two contributing so little as to be statistically insignificant.

**Table 4:** ANOVA for S/N ratios of adsorption capacity (Qe).

Source	DF	SS	MS	F	p-value	Remarks
A	4	26.00	6.50	5.85	0.02	Significant
B	4	0.65	0.16	0.36	0.96	Insignificant
C	4	7.80	1.95	0.85	0.28	Insignificant
D	4	518.13	129.53	152.17	0.00	Significant
Residual Error	8	10.25	1.28			
Total	24	562.85				

*DF* the degrees of freedom, *SS* the sum of squares, *MS* the mean squares, *F* Fischer's test, *p-value* the critical probability.

#### Analysis of residual plots

**Figure 8:**
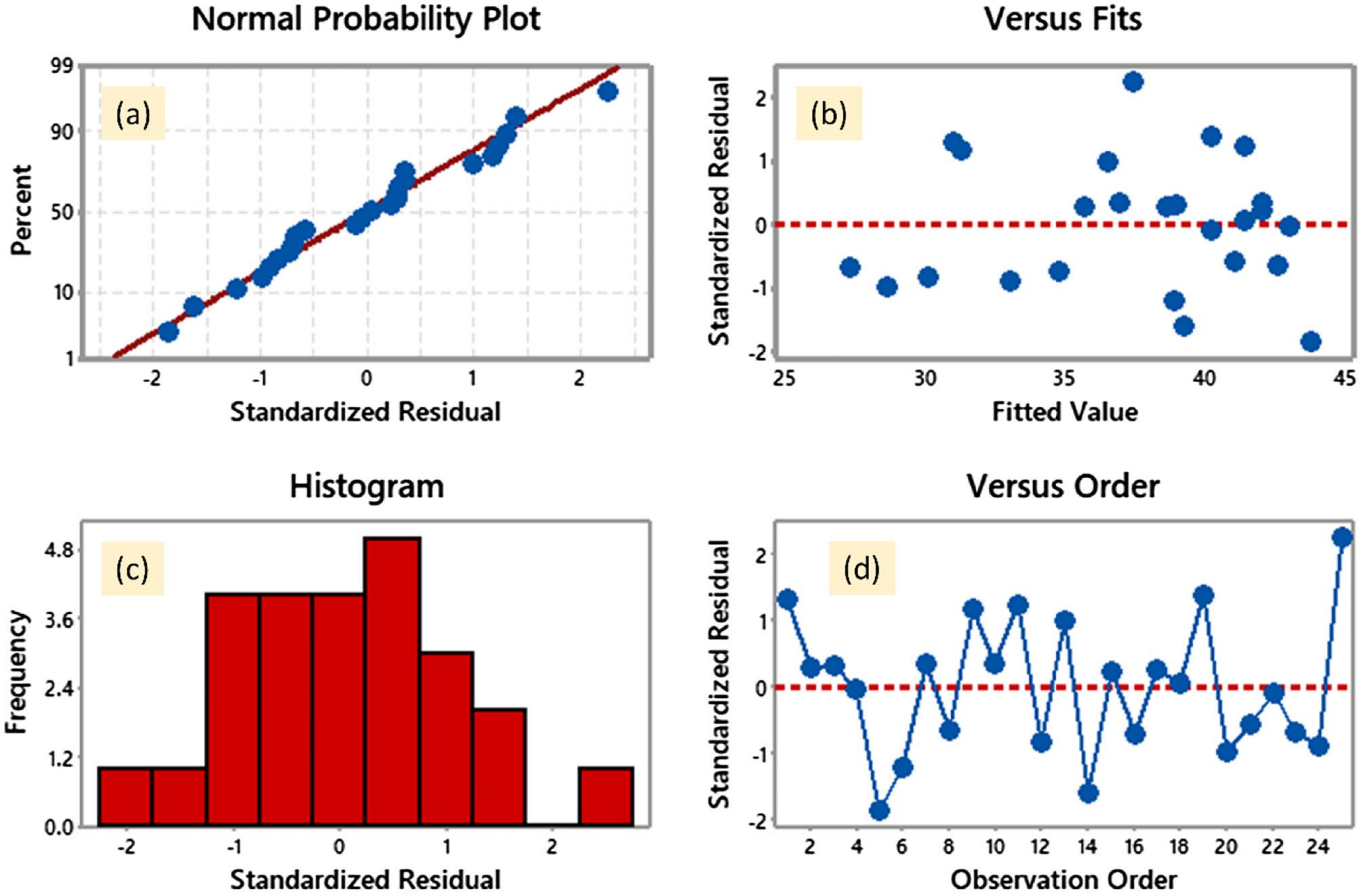
Residual plots for S/N ratios on normal probability (**a**), versus fits (**b**), histogram (**c**), and versus order (**d**).

Figure 8 displays the residual plots (normal probability plot versus fits, a histogram, and versus order) for the S/N ratios of adsorption capacity (Qe). Throughout the adsorption process, residuals follow a normal distribution, as seen by the produced normal probability plot, in which most points lie on or very close to the line. A residual vs. fitted values graphic is used to determine whether the input parameters impact the output results. This shows that the residuals have a constant variance significance^41,42^, since the spots lower in the graph are gradually horizontally oriented, while the spots higher in the graph are directed more arbitrarily toward the residual lines (zero value). The histogram bars show that the residuals only have a variance point for a small number of observations. Lastly, an examination of residuals vs. order shows that the residuals are scattered randomly with respect to the zero lines, emphasizing that they rely heavily on dye removal.

#### Fitted plots assessment

**Figure 9:**
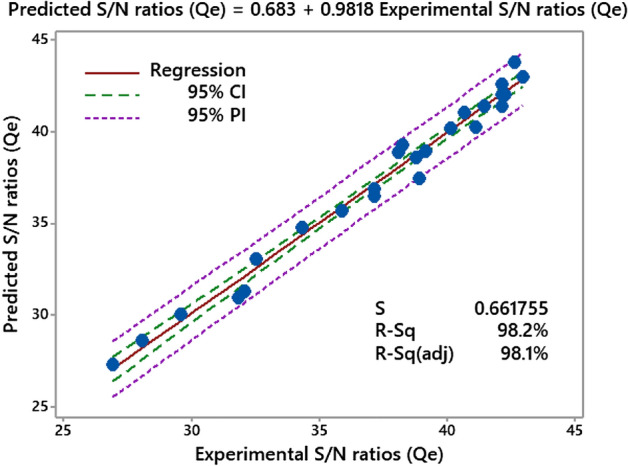
Fitted lines for the experimental S/N ratios (T%) and predicted S/N ratios (T%).

The regression equation allows for accurate computation and comparison of predicted values depending on experimental conditions^43^. Figure 9 displays the predicted vs. experimental responses in a fitted plot for the Qe of methyl orange. It can be seen that the experimental and predicted Qe are very well aligned, with an R^2^ value of 98.2% and an adjusted R^2^ of 98.1%. When there is less dispersion in the values around the mean, it is clear that the model can make an accurate prediction. The Pearson correlation between the predicted and actual Qe was 0.9818, and the study's P-value was 0.000. There is a high correlation between the predicted and observed fixation rates, as shown by this finding^44^.

#### Confirmation test

The Taguchi methodology relies on a confirmation test to further validate its findings, and this test is strongly recommended for statistical procedures. The main purpose of this validation test is to confirm the reliability of the assessments and the results^45^. Table 5 displays the results of the validation tests. In order to ensure that the process has been optimized, it is necessary first to infer the ideal circumstances. The predicted values were calculated algorithmically. In light of this, the experiment was conducted using the ideal parameters, and it was found that the S/N ratio had been adequately increased. The major objective of this study was to raise the Qe (which was achieved by an increase in the S/N ratio of 0.96). These results demonstrated the efficacy of a standardized, statistical approach to experimentation in enhancing performance.

**Table 5:** Results of the confirmation experiment.

Conditions	Initial parameters	Prediction	Confirmation experiment
Level	A2B2C3D4	A1B5C5D4	A1B5C5D4
Qe	130.12	143.134	145.23
S/N	42.28	44.09	43.24
Improvement in the S/N ratio		0.96	

### Adsorption kinetics

**Figure 10:**
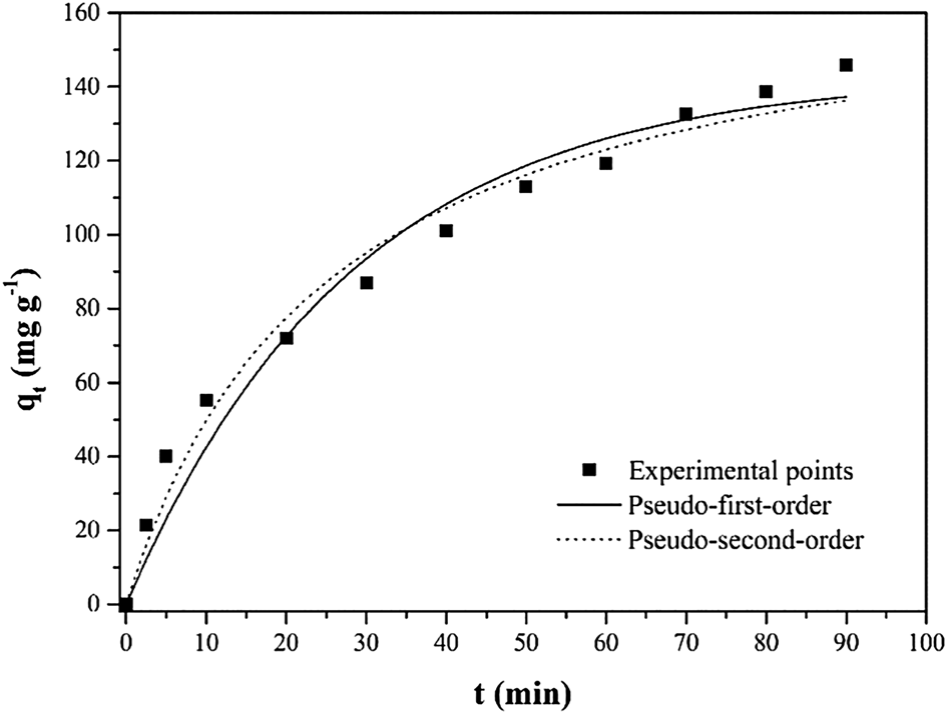
Pseudo-first-order and pseudo-second-order kinetic models for the MO adsorption on 1.5% PEG-GO/CS.

Adsorption kinetics as a function of contact time is an essential indicator for determining the rate constant of the whole adsorption process^46,47^. Hence, the adsorption kinetics of MO onto 1.5% PEG-GO/CS were studied, and the findings are shown in Fig. 10. Initial adsorption rates were high but gradually decreased as the experiment progressed, eventually reaching equilibrium. Dye adsorption was shown to be rapid during the first 20 min, and equilibrium was attained after 60 min. This rapid dye removal during the first adsorption stage has been attributed to several empty sites dispersed over the composite surfaces. Nevertheless, the dye removal rate significantly slows down in the last phase, likely owing to the significantly reduced number of empty sites on the surfaces of 1.5% PEG-GO/CS and the aggregation of these particles during adsorption. The findings show that PEG inclusion into GO/CS matrix improved adsorption performance by providing active sites for MO removal. In addition, the kinetics of adsorption were analyzed using nonlinear pseudo-first-order (PFO) (Eq. 4) and pseudo-second-order (PSO) (Eq. 5) models^48^.

4 $$ q_{t} = q_{e} \left( {1 - e^{{ - k_{1} t}} } \right) $$

5 $$ q_{t} = \frac{{k_{2} q_{e}^{2} t}}{{1 + k_{2} q_{e} t}} $$

where q_t_ (mg/g) is the maximum amount of dye that may be adsorbed in a given amount of time, t, k_1_ (min^−1^), and k_2_ (g/mg/min) are the pseudo-first and pseudo-second-order kinetic rate constants, respectively.

The fitted curve is shown in Fig. 10, and the corresponding fitted values are shown in Table 6. There is a clear preference for PSO kinetic model, as shown by the high coefficient of determination (R^2^ = 0.9810) and the presence of a good agreement between the predicted and observed qe values while analyzing the adsorption performance of MO. Besides, the PSO model presented lower average relative error (ARE) and standard deviation (SD) values^49,50^. A similar kinetics trend was reported by Liu et al.^51^ and showed that the PSO model offers the best adsorption rate for methyl orange by GO-based adsorbent. Also, Huang et al.^52^ accurately fitted the methyl orange adsorption kinetics by PSO in the presence of crosslinked chitosan-based adsorbent. The findings indicate that the chemisorption mechanism was the primary adsorption process^53^. This suggests a transfer or interchange of electrons between the adsorbent's binding sites and the methyl orange molecule. Notably, the adsorption rate of MO by 1.5% PEG-GO/CS was enhanced after incorporating PEG. According to the prior investigation, incorporating PEG increased methyl orange's adsorption efficacy through PEG-modified composite adsorbents^54^. The findings of our study indicate that the utilization of the PEG ligand has the potential to augment the elevated specific surface area of the 1.5% PEG-GO/CS composite, thereby rendering it more conducive for diffusion and leading to rapid adsorption kinetics.

**Table 6:** Kinetics parameters for MO adsorption by 1.5% PEG-GO/CS.

Pseudo-first-order	
q_1_ (mg/g)	143.3
k_1_ (1/min)	0.03522
R^2^	0.9686
ARE (%)	13.16
SD (mg/g)	9.417
Pseudo-second-order	
q_2_ (mg/g)	174.2
k_2_ (g/mg min)	0.0002299
R^2^	0.9810
ARE (%)	9.666
SD (mg/g)	7.331

### Adsorption isotherms

In the process of adsorption, adsorption isotherms play a crucial role. An adsorption isotherm is formed when an adsorbate and an adsorbent are in contact with one another over a period that is long enough. There is a state of dynamic equilibrium between the adsorbate concentration at the interface and the adsorbate concentration at the solution phase^55^, leading to obtaining the maximum adsorption capacity. The Langmuir model (Eq. (6)) and the Freundlich model (Eq. (7)) are two well-known nonlinear isotherm models that are utilized to explore the adsorption behavior in the current work. Adsorption onto the surface of a homogeneous solid adsorbent is shown to occur as a monolayer by the Langmuir fitted model^52,53^, whereas adsorption onto the surface of a heterogeneous solid adsorbent is shown to occur as a multilayer by the Freundlich fitted isotherm model.

6 $$ q_{e} = \frac{{q_{max} k_{L} C_{e} }}{{1 + k_{L} C_{e} }} $$

7 $$ q_{e} = K_{F} C_{e} {\raise0.7ex\hbox{$1$} \!\mathord{\left/ {\vphantom {1 n}}\right.\kern-0pt} \!\lower0.7ex\hbox{$n$}} $$

where q_max_ is the maximum MB adsorption capacity (mg/g), KL is the Langmuir equation constant, and Ce is the equilibrium solution concentration. The value of 1/n is used to illustrate the adsorption intensity, while the value of KF represents the Freundlich constant.

**Figure 11:**
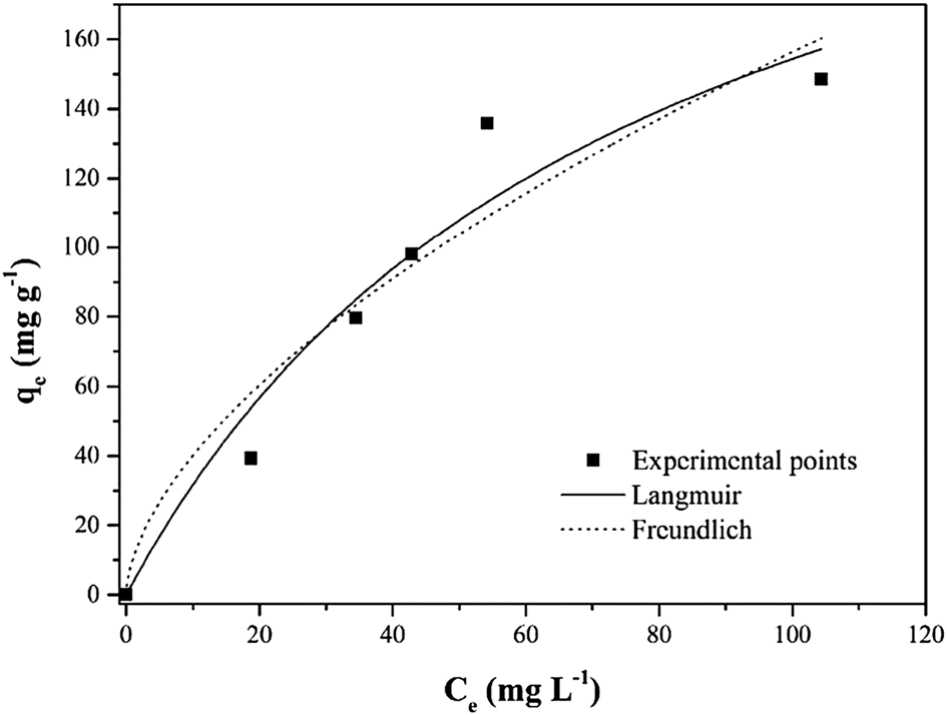
The adsorption isotherms curve of MO by 1.5% PEG-GO/CS.

Figure 11 depicts the fitted curve, while Table 7 provides the obtained isotherm parameters. The adsorption isotherms for 1.5% PEG-GO/CS showed monolayer adsorption on the homogeneous adsorbent surface, as shown by a high coefficient of determination (R^2^) found for the Langmuir model. The Langmuir model also presented lower values of ARE and SD^56^. The adsorption isotherms for 1.5% PEG-GO/CS showed monolayer adsorption on the heterogeneous adsorbent surface, as shown by high correlation co-efficiency (R^2^). The findings show that the 1.5% PEG-GO/CS can adsorb up to 271.00 mg/g of MO. As a larger KL of the adsorbent leads to a better adsorption performance at a low concentration^57^, this suggests that the 1.5% PEG-GO/CS significantly boosted the adsorption capacity of MO. According to Table 8, when comparing our adsorbent to other adsorbents for MO adsorption, 1.5% PEG-GO/CS has an outstanding adsorption capacity, much greater than others. This demonstrates that the 1.5% PEG-GO/CS has great potential as an ideal adsorbent for cleaning up wastewater containing MO.

**Table 7:** Adsorption isotherms parameters for MO.

Langmuir	
q_max_ (mg/g)	271.0
K_L_ (L/mg)	0.01324
R^2^	0.9486
ARE (%)	13.29
SD (mg/g)	16.63
Freundlich	
K_F_ (mg/g)(L/mg)^−1/nF^	10.29
1/n	0.5909
R^2^	0.9226
ARE (%)	16.79
SD (mg/g)	20.41

**Table 8:** A comparison of the MO adsorption capacity with the previously reported literature.

Adsorbent	pH	Kinetics	Isotherms	*q*_max_ (mg/g)	References
Functionalized graphene oxide aerogel	3	Pseudo-second-order kinetic	Langmuir isotherm	202.8	^58^
Chitosan	4	Pseudo-second-order kinetic	Langmuir isotherm	34.83	^59^
Protonated cross-linked chitosan	1.0–9.1	Pseudo-second-order kinetic	Langmuir isotherm	89.30	^52^
Konjac glucomannan/GO	7	Pseudo-second-order kinetic	Freundlich isotherm	51.6	^60^
Chitosan/alumina	6	Pseudo-second-order kinetic	Langmuir isotherm	33	^61^
γ-Fe_2_O_3_ crosslinked chitosan	6.6	Pseudo-second-order kinetic	N/A	29.46	^62^
Goethite	3	Pseudo-second-order kinetic	Langmuir isotherm	55	^63^
Chitosan beads	3	Pseudo-second-order kinetic	Langmuir isotherm	73	^63^
Goethite/chitosan beads	3	Pseudo-second-order kinetic	Langmuir isotherm	84	^63^
Iron(II) cross-linked chitin-based gel beads	6.7 ± 0.1	N/A	Langmuir isotherm	107.5	^64^
Multiwalled carbon nanotubes	2	Pseudo-second-order kinetic	Langmuir isotherm	35.4–64.7	^65^
Alkali-Activated Multiwalled carbon nanotubes	7	Pseudo-second-order kinetic	Freundlich isotherm	149	^66^
1.5% PEG-GO/CS	2	Pseudo-second-order kinetic	Langmuir isotherm	271	This work

### Ionic strength and reusability

**Figure 12:**
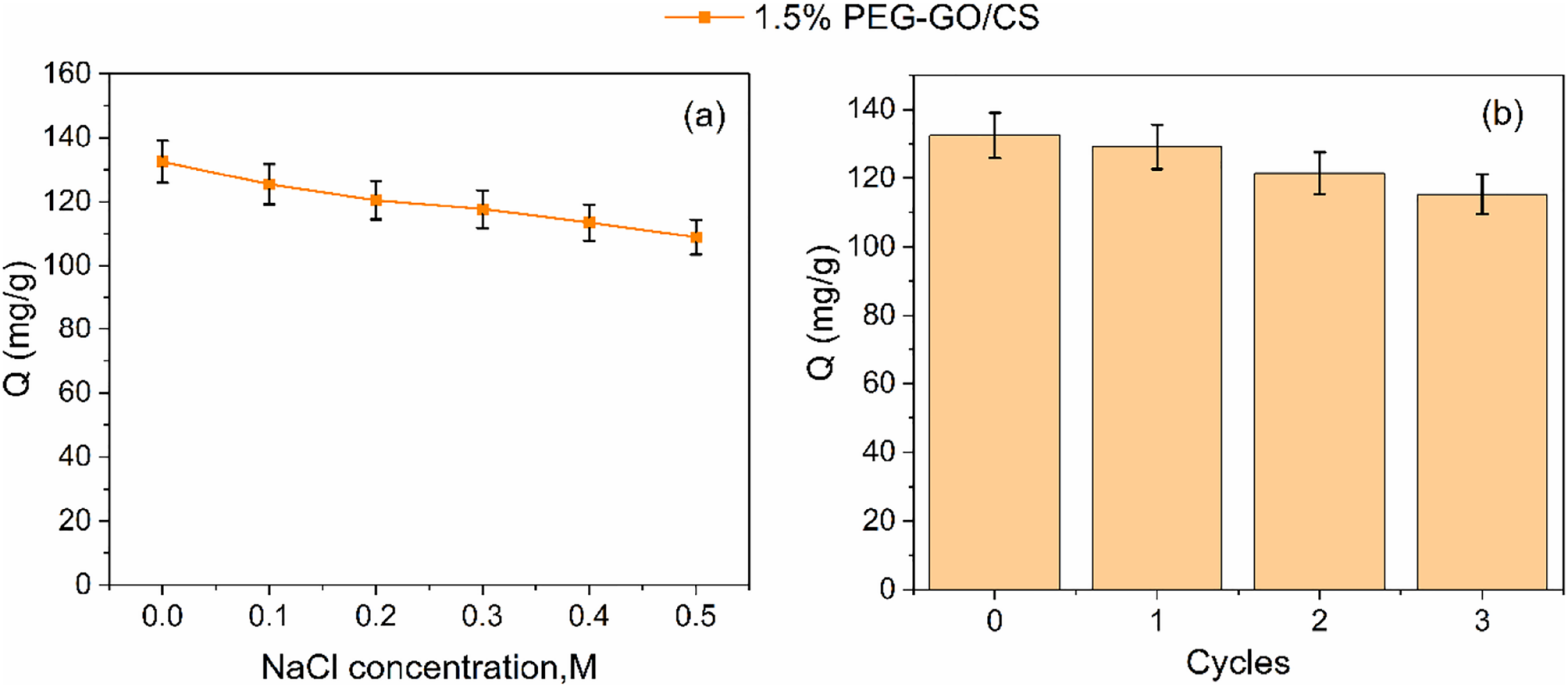
(**a**) Effect of NaCl on MO adsorption capacity and (**b**) recyclability tests by 1.5% PEG-GO/CS, respectively.

Wastewater from industries may comprise various pollutants, including dissolved and suspended compounds, acids or alkalis, salts, metal ions, and more. The presence of ions increases the ionic strength of a solution, which may affect adsorption efficiency^67^. NaCl was introduced to the solution at different concentrations to investigate the effect of ionic strength on the adsorption capacity of MO in the 1.5% PEG-GO/CS. The adsorption capability of MO was shown to decrease in tandem with increasing NaCl content (Fig. 12a). Low adsorption capacity at high ionic strength may be caused by the salt's ability to shield the adsorbent surface from the electrostatic attraction between ions with opposing charges^68^.

Commercially available sorbents must be recyclable and capable of several reuses to remain cost competitive^69^. For this purpose, additional studies were conducted using 1.5% PEG-GO/CS for up to three cycles under optimal conditions. The findings show that after 3 cycles, the 1.5% PEG-GO/CS lost just 12% of adsorption capacity, showing that the PEG-modified GO/CS composite is recyclable and reusable (Fig. 12b). The superior performance of the 1.5% PEG-GO/CS is attributed to its positive surface charge and enriched functional groups adsorption binding sites.

### Adsorption mechanism

**Figure 13:**
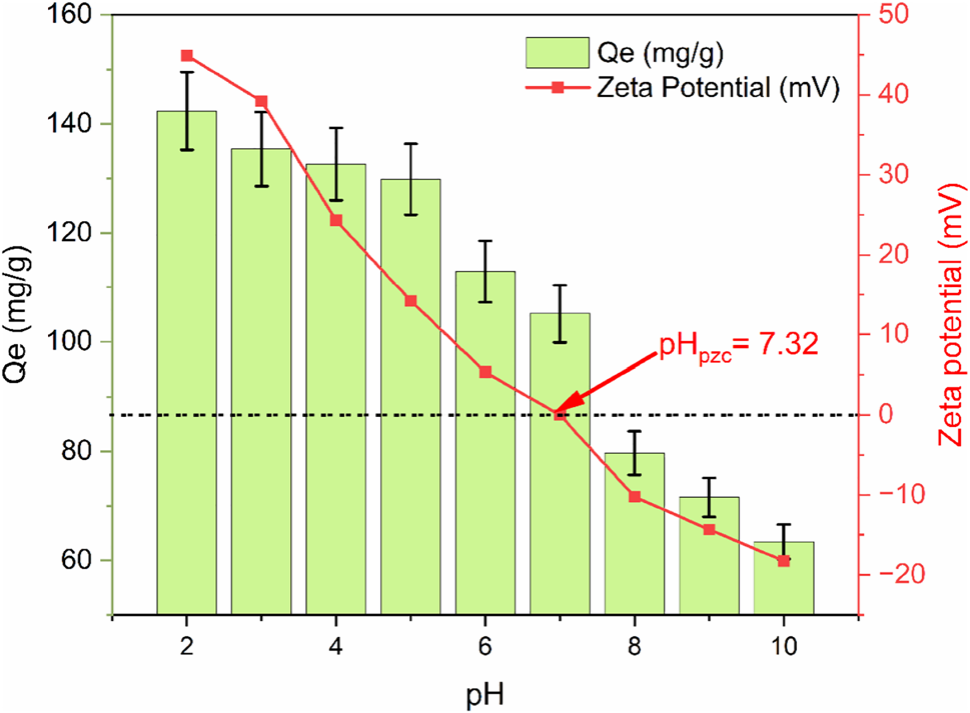
The effect of the pH on the MO adsorption capacity and zeta potential.

**Figure 14:**
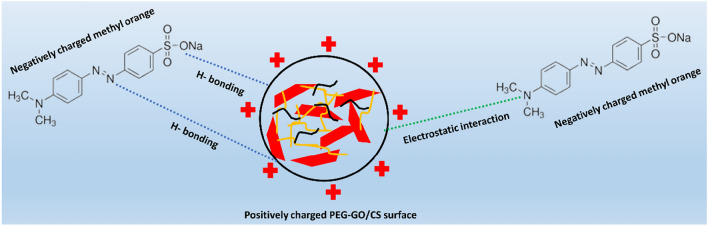
Proposed MO adsorption mechanism on PEG-GO/CS.

Considering that the surface charge of both the adsorbent and the adsorbate is very sensitive to pH values, it has been suggested that the pH of the solution is crucial for adsorption efficiency. The results of testing the MO adsorption capability in the presence of 1.5% PEG-GO/CS composite at pH values ranging from 2 to 10 are shown in Fig. 13. It was noticed that the MO adsorbed by 1.5% PEG-GO/CS decreases when the pH increases; at pH 10 the removal efficiency decreases dramatically. In particular, the highest adsorption capacity of MO was 142 mg/g at pH 2, which is in line with previously published research^70^. According to the data presented in Fig. 12, it can be observed that the composites of 1.5% PEG-GO/CS exhibit positive surface charge, with a pH value corresponding to the point of zero charge (pH_PZC_) of 7.32. At pH values below the pH_PZC_, the adsorption of MO anions is higher due to strong electrostatic attraction compared to pH values above pH_PZC_^71^. To some extent, after MO has been diluted in water, the sulfonate group in the MO dye (R-SO_3_Na) breaks apart to create anionic dye ions, which then bind to the cationic sites of the adsorbent through electrostatic attraction. On the other hand, adsorption capability falls when the pH of the MO solution rises. As the PEG-GO/functional CS's groups are deprotonated in a basic solution, the dye-uptake capacity drops along with it^72^. This is because the interaction between the PEG-GO/CS and MO is attenuated. The MO adsorption capability may decrease because the numerous OH ions compete with the MO anions for adsorption sites. Overall, it is considered that electrostatic interactions, π–π, and H-bond are mainly responsible for MO adsorption by PEG-GO/CS composite (Fig. 14)^73^.

## Conclusions

This research describes the hydrothermal synthesis of a composite of graphene oxide (GO) and chitosan (CS) functionalized with polyethylene glycol (PEG) and used to remove methyl orange (MO) dye from water. The characterization studies indicated that adding PEG-tuned GO/CS composite surface structures improved sorption performance. The composite with 1.5% PEG-GO/CS exhibited a robust structure and a high removal efficiency (71%) compared to the others. The effects of solution pH, contact duration, adsorbent dose, and starting dye concentration on the dye adsorption process were studied. The L_25_ Taguchi experimental design approach was used to get these ideal values for the variables above. These parameters were found to be most favorable at an initial pH of 2, a period of 90 min, an adsorbent dosage of 10 mg/10 mL, and an initial MO concentration of 200 mg/L. Compared to prior investigations, the adsorption capacity of the composite was higher at 271 mg/g, and it showed superior agreement with the pseudo-second-order kinetic model and the Langmuir isotherm. The surface charge of 1.5% PEG-GO/CS was highly positive, which may assist in improving the sorption capacity of MO from an aqueous solution. NaCl, representing ionic strength, was shown to negatively impact the adsorption capacity. The 1.5% PEG-GO/CS composites can be regenerated and reused for three consecutive cycles. The high dependence of sorption capacity on pH suggested that the electrostatic interaction was the primary driving mechanism for MO sorption.

### Supplementary Information

Supplementary Information.

## Data Availability

The datasets generated during the current study are available from the corresponding author on reasonable request (Prof. Yingjie Cai, Y. Cai).

## Code availability

No code was attempted or used during the current manuscript.
