# Supplementary material for: Tuning the surface functionality of polyethylene glycol-modified graphene oxide/chitosan composite for efficient removal of dye
Source: Sci Rep. 2023 Aug 18;13:13460. doi: 10.1038/s41598-023-40701-9 (PMC10439132; doi:10.1038/s41598-023-40701-9)
Supplement: Supplementary file 1 — Supplementary Information. [file 41598_2023_40701_MOESM1_ESM.docx]

**Supporting Information**

**Tuning the surface functionality of polyethylene glycol-modified graphene oxide/chitosan composite for efficient removal of dye**

Md. Nahid Pervez^1,2,4#^, Md Anwar Jahid^1#^, Mst. Monira Rahman Mishu^3^, Md Eman Talukder^1^, Antonio Bounerba^3^, Tao Jiang^4^, Yanna Liang^4^, Shuai Tang^5^, Yaping Zhao^5^, Guilherme L. Dotto^6^, Yingjie Cai^1*^, Vincenzo Naddeo^2*^

*^1^Hubei Provincial Engineering Laboratory for Clean Production and High Value Utilization of Bio-Based Textile Materials, Wuhan Textile University, Wuhan, 430200, China*

*^2^Sanitary Environmental Engineering Division (SEED), Department of Civil Engineering, University of Salerno, via Giovanni Paolo II 132, 84084 Fisciano (SA), Italy*

*^3^Department of Chemistry and Biology “Adolfo Zambelli”, University of Salerno, 84084 via Giovanni Paolo II, Fisciano, Italy*

*^4^Department of Environmental and Sustainable Engineering, University at Albany, State University of New York, Albany, NY, 12222, USA*

*^5^Shanghai Engineering Research Center of Biotransformation of Organic Solid Waste, School of Ecological and Environmental Sciences, East China Normal University, and Institute of Eco-Chongming, Shanghai, 200241, China*

*^6^Research Group on Adsorptive and Catalytic Process Engineering (ENGEPAC), Federal University of Santa Maria, Av. Roraima, 1000-7, 97105-900 Santa Maria, RS, Brazil*

*# These authors contributed equally to the work*

**^⁎^ Corresponding authors:**

yingjiecai@wtu.edu.cn (Y. Cai); vnaddeo@unisa.it (V. Naddeo)

1% GO/CS

|  |  |  |
| --- | --- | --- |
| 1.5% PEG-GO/CS | | |
|  |  |  |
| 2% PEG-GO/CS | | |
|  |  |  |
|  | | |

**Figure S1.** Core levels XPS spectra.

**Table S1.** Summary of binding energy and atomic surface concentration of GO/CS, 1.5 % PEG-GO/CS and 2 % PEG-GO/CS.

| Samples |  | Binding energy (eV) |  |  |  |  |  |  |
| --- | --- | --- | --- | --- | --- | --- | --- | --- |
|  | O1s |  | C1s |  | N1s |  | Cl2p | Si2p |
| GO/CS | 531.05 | 531.12 | 284.80 | 284.89 | 408.54 |  |  |  |
| 1.5% GO/CS/PEG | 532.51 | 532.65 | 284.80 | 285.23 | 399.92 | 400.93 | 200.94 | 102.36 |
| 2% GO/CS/PEG | 531 | 531.06 | 284.63 | 284.80 | 397.56 |  |  | 100.75 |
| Atomic concentration (%) |  |  |  |  |  |  |  |  |
|  | O |  | C |  | N |  |  |  |
| GO/CS | 33.57 |  | 63.47 |  | 0.82 |  |  |  |
| 1.5% GO/CS/PEG | 35.71 |  | 62.71 |  | 2.36 |  |  |  |
| 2% GO/CS/PEG | 34.93 |  | 65.21 |  | 1.22 |  |  |  |

| mV= 39.7 |
| --- |
| (a) 1% GO/CS |
| mV= 44.9 |
| (b) 1.5% PEG-GO/CS |
| mV= 41.5 |
| (c) 2% PEG-GO/CS |

**Figure S2.** Zeta potential values.
